# Supplementary material for: Transcriptomic Immune Profiles Can Represent the Tumor Immune Microenvironment Related to the Tumor Budding Histology in Uterine Cervical Cancer
Source: Genes (Basel). 2022 Aug 7;13(8):1405. doi: 10.3390/genes13081405 (PMC9407871; doi:10.3390/genes13081405)
Supplement: Supplementary file 1 [file genes-13-01405-s001.zip › Figure S1_features of high tumor budding histology.pdf]

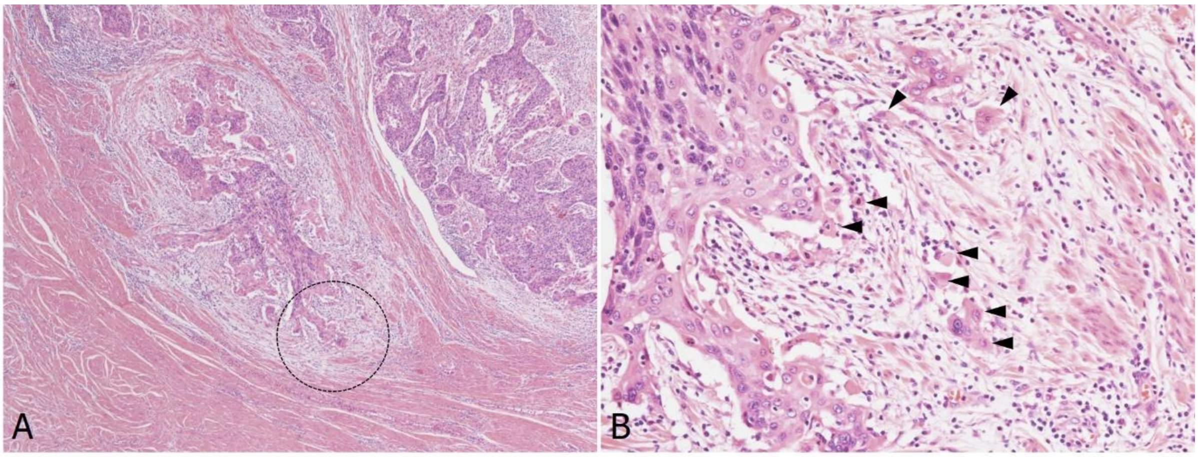


**Figure S1**. **Representative microscopic features of high tumor budding (TB) histology**. (A) Tumor buddings were identified at the advancing edge (peritumoral) of invasive cancer. (B) On the high magnified lesion (black dotted circle in A), each TB histology was particularly indicated by black arrowheads; the TB count was compatible with high TB (All, hematoxylin and eosin stain; original magnification, A, 40x; B, 200x).
