# Supplementary figures and images for: Transcriptomic Immune Profiles Can Represent the Tumor Immune Microenvironment Related to the Tumor Budding Histology in Uterine Cervical Cancer
Source: Genes (Basel). 2022 Aug 7;13(8):1405. doi: 10.3390/genes13081405 (PMC9407871; doi:10.3390/genes13081405)

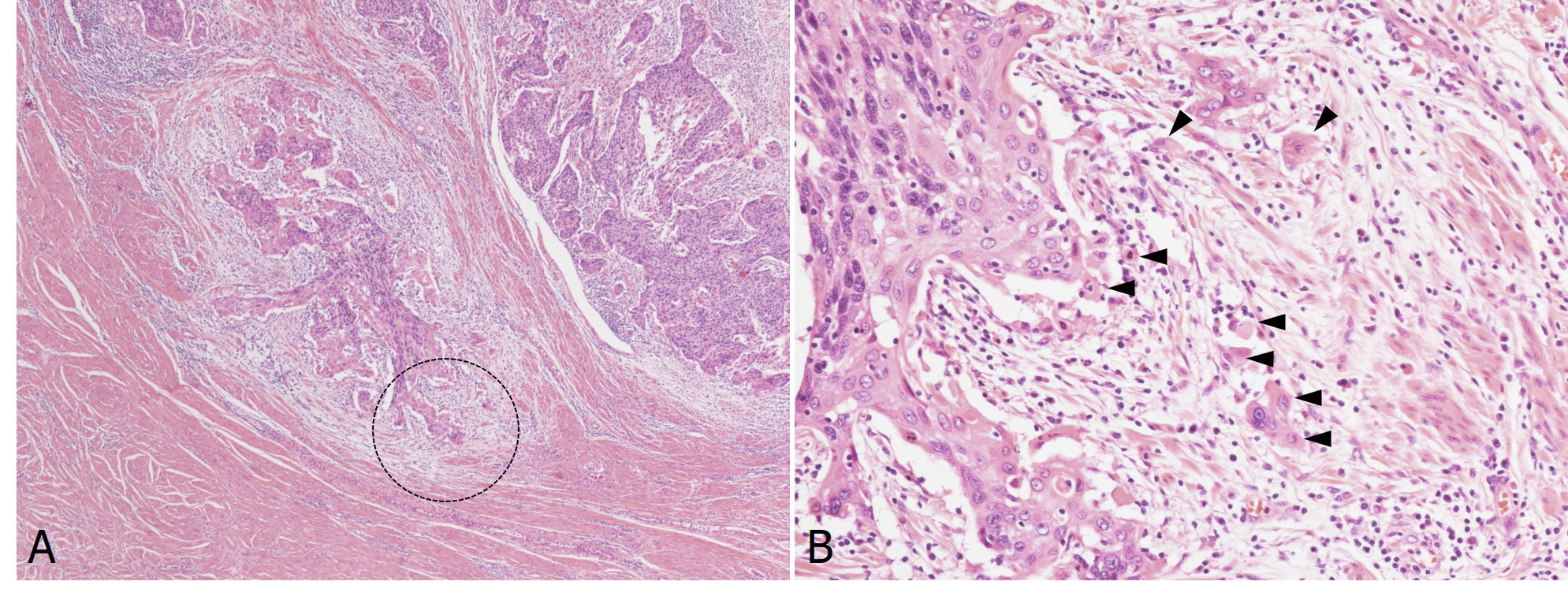

Supplement: Supplementary file 1 [file genes-13-01405-s001.zip › Figure S1.tif]
